# FEASIBILITY OF CARE PARTNER-ASSISTED CONTINUOUS GLUCOSE MONITORING IN OLDER CHINESE AMERICANS WITH MCI 

**DOI:** 10.1093/geroni/igaf122.1416b

**Published:** 2025-12-31

**Authors:** Yaguang Zheng, Richard Lipton, Katharine Lawrence, Susan Zweig, Linda Siminerio, Jessica Zwerling, Eric Chen, Bei Wu

**Affiliations:** New York University, New York, New York, United States; Albert Einstein College of Medicine, Bronx, New York, United States; NYU Grossman School of Medicine, New York, New York, United States; NYU Grossman School of Medicine, New York, New York, United States; University of Pittsburgh, Pittsburgh, Pennsylvania, United States; Albert Einstein College of Medicine, Bronx, New York, United States; New York University, New York, New York, United States; NYU Shanghai, Shanghai, China (People's Republic)

## Abstract

Mild cognitive impairment (MCI) can increase care burden for type 2 diabetes (T2D), and has been evident in the older Chinese Americans. Continuous glucose monitoring (CGM) offers a promising solution but is underutilized in this population, we thus aimed to examine the feasibility of using CGM among them. Older Chinese Americans with MCI and T2D (N=11) wore a CGM for 10 days and real-time CGM data were shared with their care partners for immediate support of their diabetes management. Both participants and their care partners received education on CGM data use. Additionally, care partners assisted patients in completing a diary for daily food intake, physical activity, and antihyperglycemic medication. Older Chinese Americans with MCI and T2D had a mean age of 74.5 ± 5.2 years, with 63.6% female, 100% retired, 36.4% college education, and 90.9% family income<$30,000. The care partners were 59.4 ± 20.6 years old, 63.6% female, 54.5% retired, and 54.5% college education. The relationship of care partners to participants was couples (45.5%) and adult children (36.4%). The primary language was Chinese. Most of them (90.9%) had no prior experience using CGM. All participants adhered to the 10-day CGM use with the retention rate of 100%. Most participants (90.1%) indicated they would like to continue receiving CGM-guided intervention. Participant enrollment and data collection were completed in two months. CGM use in care partner-assisted diabetes management among older Chinese Americans with MCI is feasible. Findings are informative for applying CGM interventions in this population to address their care burden.

